# The relationship of malaria between Chinese side and Myanmar’s five special regions along China–Myanmar border: a linear regression analysis

**DOI:** 10.1186/s12936-016-1413-4

**Published:** 2016-07-18

**Authors:** Jian-Wei Xu, Hui Liu

**Affiliations:** Yunnan Institute of Parasitic Diseases, Yunnan Provincial Centre of Malaria Research, Yunnan Provincial Collaborative Innovation Centre for Public Health and Disease Prevention and Control, Yunnan Provincial Key Laboratory of Vector-borne Diseases Control and Research, 665000 Puer, China

**Keywords:** Malaria, Linear regression analysis, Chinese-Myanmar border

## Abstract

**Background:**

Understanding malaria along the international border of two countries is important for malaria control and elimination; however, it is difficult to investigate a quantitative relationship between two countries’ border areas due to a shortage of malaria surveillance data.

**Methods:**

A linear regression analysis was conducted to investigate the logarithmic annual parasite incidence (API), numbers of imported cases and local infections in 19 Chinese border counties, with logarithmic API and parasitic prevalence in Myanmar’s five special regions.

**Results:**

API in 19 Chinese counties was stronger correlated with parasite prevalence than with API in five special regions of Myanmar, correlation coefficient (R) 0.8322 (95 % CI 0.0636–0.9084) versus 0.9914 (95 % CI 0.9204–0.9914). Numbers of imported malaria cases and local malaria infections in 19 Chinese counties were also closer correlated with parasite prevalence than with API in five special regions of Myanmar.

**Conclusions:**

There is a strong correlation of malaria between China’s side and Myanmar’s side along the international border. Parasite prevalence is a better indicator of the true malaria situation in a setting without sound surveillance and reporting system. China should reconsider its definition of imported malaria which neglects imported malaria by mosquitoes and asymptomatic parasite carriers.

**Electronic supplementary material:**

The online version of this article (doi:10.1186/s12936-016-1413-4) contains supplementary material, which is available to authorized users.

## Background

Malaria control has achieved remarkable progress over the last 15 years [1]. By adopting the World Health Organization (WHO) Global Technical Strategy for Malaria 2016–2030, WHO Member States have endorsed the bold vision of a world free of malaria, and set the ambitious new target of reducing the global malaria burden by 90 % by 2030 [2]. In the Greater Mekong Subregion (GMS), the goal is to eliminate *Plasmodium falciparum* malaria by 2025 and to eliminate malaria by 2030 in all countries [3]. However, according to WHO estimates, there were 214 million new cases of malaria worldwide in 2015 (range 149–303 million). The number of malaria cases in Southeast Asia Region (10 %) ranked the second and followed African Region (88 %) [1]. GMS nations still face great challenges. Malaria epidemiology in this region exhibits enormous geographical heterogeneity and the disease is concentrated mainly in remote areas [3, 4]. Resistance of *P. falciparum* to anti-malarial drugs, including resistance to artemisinin-based combination therapy (ACT), is of great concern [3]. China has made substantial progress towards elimination and aims to finally eliminate malaria on its border areas of Southern China by 2020 [5]. Myanmar remains the country with the highest burden malaria in the GMS [3]; however, there is not sufficient consistent data to evaluate the country’s malaria trends for the 2000–2015 period [1].

In order to develop sound malaria control and elimination strategies, it is necessary to understand the status of malaria between two countries along their international border. However, it is difficult to investigate the quantitative statistics between the two countries’ border areas because of lack of malaria surveillance data from similar monitoring systems. During 2007–2013, with the support of the sixth and tenth rounds of China’s Global Fund to Fight AIDS, Tuberculosis and Malaria (GFATM), more intensive malaria surveillance was carried out on China–Myanmar border areas [6]. This enabled an analysis of the quantitative relationship of malaria between the Chinese and Myanmar border areas. Linear regression analysis is the most commonly used technique to investigate two quantitative variables [7]. Here, a linear regression was used to analyse malaria surveillance data between the Chinese and Myanmar border areas from 2008 to 2013.

## Methods

### Study site and population

China and Myanmar share a border of 2185 km. Like most parts of the GMS, *Anopheles minimus* and *Anopheles dirus* are the primary inland malaria vectors along China–Myanmar border. Other less efficient vectors are associated with rice fields. *Anopheles minimus* survives in light forest and in foothills after deforestation. *Anopheles dirus* only survives in shaded and humid areas [8]. Malaria transmission occurs the whole year in lowlands and foothills below 800–1500 m, but the peak occurs during the rainy season from May to November each year [9].

The border is extremely porous. Chinese official border crossing stations recorded 10,999,677 person-times of border crossings in 2009. Political isolation has resulted in a lack of governmental health service structure in the five special regions of Myanmar [10].

**Fig. 1:**
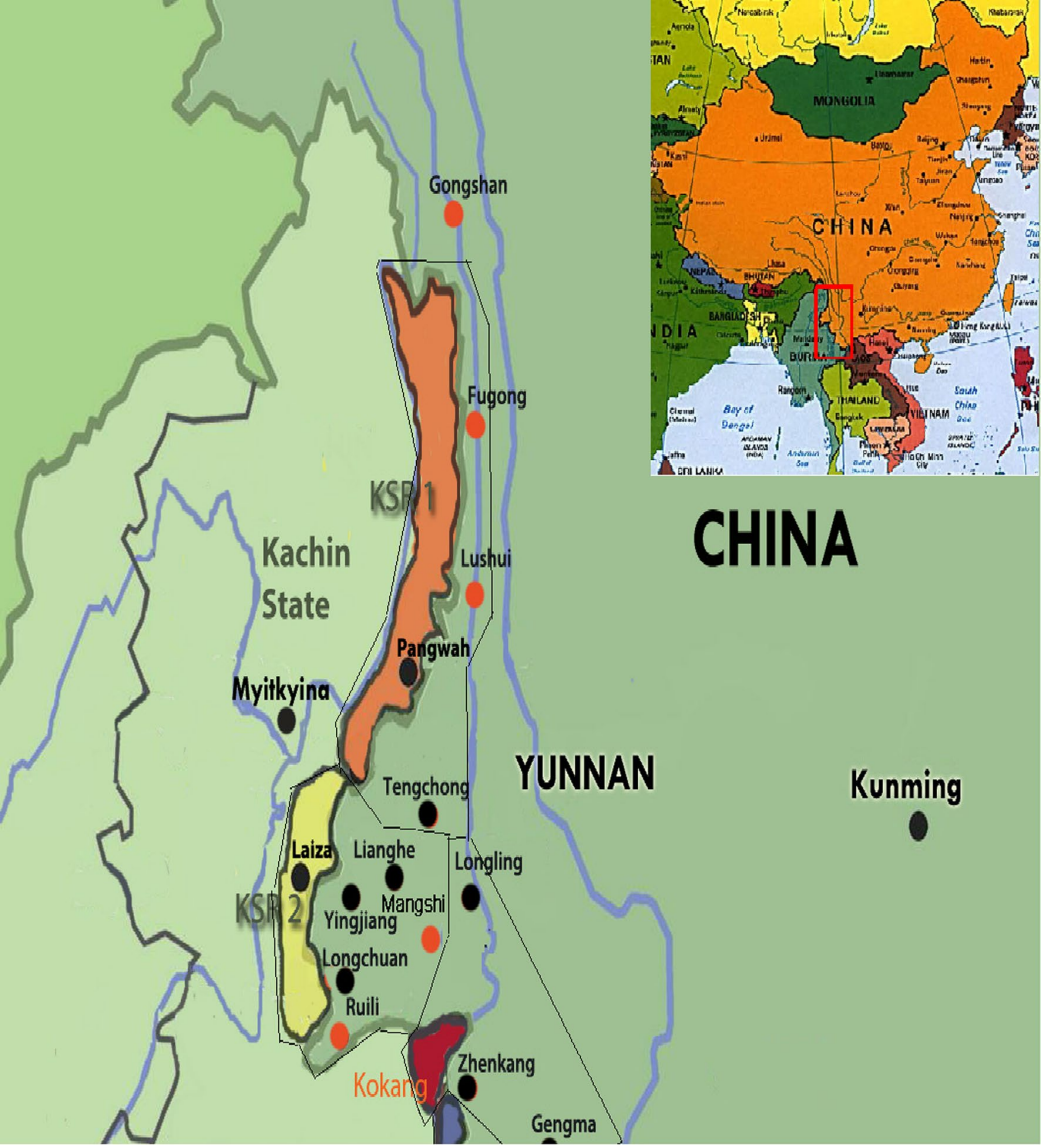
Map of study site and neighbouring region. 19 Chinese border counties: Gongshan (GS), Fugong (FG), Lushui (LS), Tengchong (TC), Longling (LL), Longchuan (LC), Yingjiang (YJ), Lianghe (LH), Ruili (RL), Mangshi (MS), Zhenkang (ZK), Gengma (GM),Cangyuan (CY), Ximeng (XM), Menglian (ML), Lancang (LC), Menghai (MH), Jinghong (JH) and Mengla (ML). Five Myanmar’s special regions: Kachin Special Region I (KSR1), Kachin Special Region II (KSR2), Kokang, Shan Special Region II (Wa) and Shan Special Region IV (SR4)

From July 2007 to December 2013, the sixth and tenth rounds of China’s GFATM programmes covered 19 Chinese counties and five Myanmar special regions along the China–Myanmar border (Fig. 1). The population of the study comprised 4,687,896 residents in 19 border counties in China and 586,000 in the five special regions in Myanmar. The GFATM programmes established malaria diagnoses and treatment stations to test for malaria parasites by microscopy in the five special regions of Myanmar, and strengthened microscopy, especially targeting border crossers in the 19 Chinese counties [11].

### Data collection

Chinese Information System for Disease Control and Prevention (CISDCP) collected and reported upon malaria cases and related information on a daily basis, such as travel history, in order to identify the place of infection in 19 counties of China. Malaria patients who had been in Myanmar within 1 month prior to diagnosis were classified as imported malaria in CISDCP [5, 12]. The Health Information System of Health Poverty Action (HPA), which was established and maintained by the GFATM programmes, collected and reported number of malaria cases monthly from the five special regions of Myanmar. A cross-sectional survey was carried out each year to measure parasite prevalence among residents in the five special regions of Myanmar from 2008 to 2013. In total six surveys were conducted. The sixth round of GFATM programme covered four of the five special regions of Myanmar and undertook the first four surveys between January and March in 2008, 2009, 2010, and 2011, respectively. The tenth round of GFATM programme covered all five special regions and undertook the last two surveys in 2012 and 2013. With implementation of control interventions, the decreased parasite prevalence made it difficult to measure the parasite prevalence during dry season, i.e., lower malaria transmission seasons. The timing of the last two surveys was changed to between September and November in 2012 and 2013, respectively. The study design of all the six surveys was described by Wang et al. who reported the first baseline survey [10].

### Statistical analysis

First, annual parasite incidence (API) per ten-thousand person-years and parasite prevalence (%) were calculated. In order to obtain a more normal distribution of these data, the API, numbers of imported cases and local infections in 19 Chinese border counties, and parasite prevalence and API in Myanmar’s border areas were transformed into common logarithms. A linear regression analysis was then used to analyse the relationship between the API in 19 Chinese border counties, and parasite prevalence and API in the five special regions of Myanmar. The relationship between numbers of imported malaria cases and local malaria infections in Chinese border areas, and parasite prevalence and the API in Myanmar was also analysed. The analyses showed a stronger correlation between malaria in 19 Chinese border counties and parasite prevalence in the five special regions of Myanmar. The relationship between parasite prevalence in each special region of Myanmar and the API of its neighbouring counties of China was further analysed [7].

## Results

**Fig. 2:**
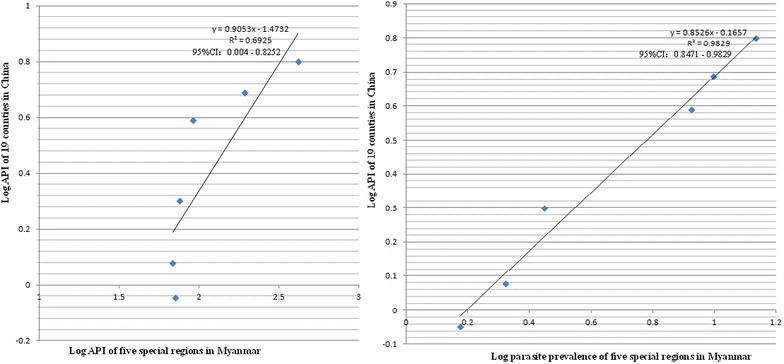
The API in 19 border counties of China and API in five special regions of Myanmar versus the API in the same areas of China and parasite prevalence of Myanmar

**Fig. 3:**
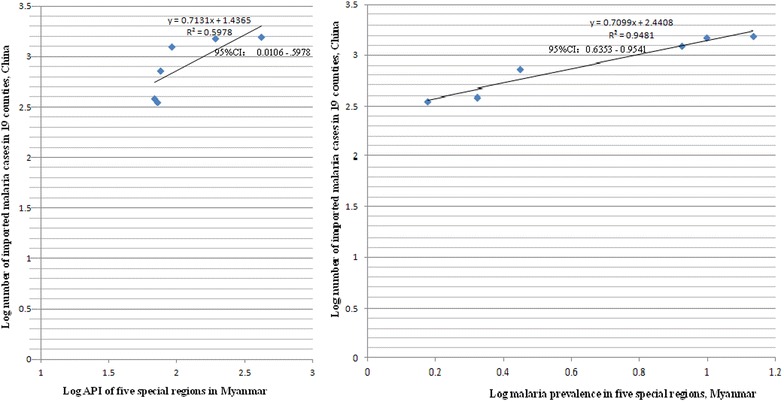
The number of imported malaria cases in 19 border counties of China and API in five special regions of Myanmar versus the number of imported malaria cases in the same areas of China and parasite prevalence of Myanmar

**Fig. 4:**
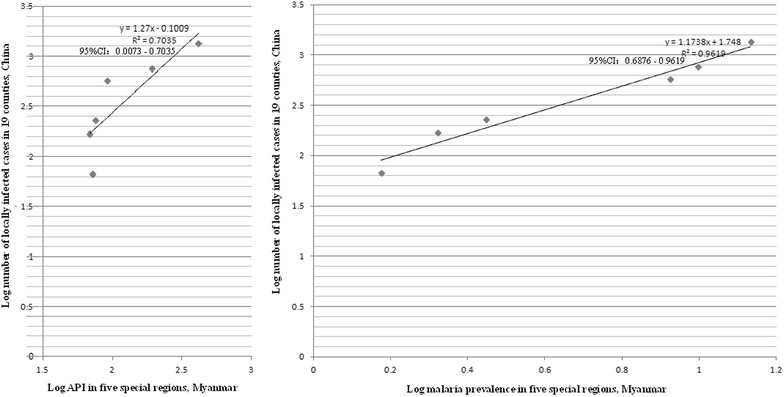
The number of local malaria infections in 19 border counties of China and API in five special regions of Myanmar versus the number of local malaria infections in the same areas of China and parasite prevalence of Myanmar

In general, the API of 19 Chinese counties was less correlated with the API of Myanmar’s five special regions (Fig. 2). Correlation coefficients (R) were 0.8322 (95 % CI 0.0636–0.9084) for *Plasmodium spp.*, 0.8147 (0.0089–0.8147) for *Plasmodium falciparum* and 0.7626 (−0.1285 to 0.7626) for *Plasmodium vivax* (Additional file 1). However, API of 19 Chinese counties was strongly correlated with parasite prevalence in five special regions of Myanmar (Fig. 2), R 0.9914 (0.9204–0.9914) for *Plasmodium spp.*, 0.9898 (0.9061–0.9898) for *P. falciparum* and 0.9693 (0.7401–0.9694) for *P. vivax* (Additional file 1). Similar results were obtained for numbers of imported malaria cases and local malaria infections in 19 counties of China (Figs. 3, 4). The number of imported malaria cases in 19 Chinese counties was closely correlated with parasite prevalence in five special regions of Myanmar (Table 1). The number of local malaria infections in 19 Chinese counties was also strongly correlated with parasite prevalence in five special regions of Myanmar (Table 1). The parasite prevalence in each special region of Myanmar also showed a close correlation with the API in its counterpart areas in China when the relationship between the parasite prevalence in each special region of Myanmar and API in its neighbouring counties of China was analysed (Table 1). This further shows that API on China side was strongly correlated with parasite prevalence on Myanmar border areas.

**Table 1:** Results of linear regression analysis for malaria between China and Myanmar border areas

Relationship	R (95 % CI)	R^2^ (95 % CI)	*P* value
Annual *P. falciparum* incidence between 19 Chinese border counties and Myanmar’s five special regions	0.8147 (0.0089–0.8147)	0.6637 (0.0001–0.6637)	<0.05
Annual *P. vivax* incidence between 19 Chinese border counties and Myanmar’s five special regions	0.7626 (−0.1285–0.7626)	0.5815 (0.0165–0.5815)	>0.05
*Annual parasite incidence (API) between 19 Chinese border counties and Myanmar’s five special regions*	*0.8322 (0.0636–0.9084)*	*0.6925 (0.004–0.8252)*	*<0.05*
Between annual *P. falciparum* incidence in 19 Chinese border counties and *P. falciparum* prevalence in Myanmar’s five special regions	0.9898 (0.9061–0.9898)	0.9797 (0.8209–0.9799)	<0.001
Between annual *P. vivax* incidence in 19 Chinese border counties and *P. vivax* prevalence in Myanmar’s five special regions	0.9693 (0.7401–0.9694)	0.9395 (0.5477–0.9397)	<0.001
*Between API in 19 Chinese border counties and parasite prevalence in Myanmar’s five special regions*	*0.9914 (0.9204–0.9914)*	*0.9829 (0.8471–0.9829)*	*<0.001*
Between the number of imported *P. falciparum* cases in 19 Chinese border counties and annual *P. falciparum* incidence in Myanmar’s five special regions	0.7863 (−0.0697–0.7863)	0.6183 (0.0049–0.6183)	>0.05
Between the number of imported *P. vivax* cases in 19 Chinese border counties and annual *P. vivax* incidence in Myanmar’s five special regions	0.6940 (−0.2692–0.6940)	0.4816 (0.0725–0.4816)	>0.05
*Between the number of imported malaria cases in 19 Chinese border counties and API in Myanmar’s five special regions*	*0.7732 (−0.1030 –0.7732)*	*0.5978 (0.0106–0.5978)*	*>0.05*
Between the number of imported *P. falciparum* cases in 19 Chinese border counties and *P. falciparum* prevalence in Myanmar’s five special regions	0.9768 (0.7971–0.9768)	0.9541 (0.6353–0.9541)	<0.001
Between the number of imported *P. vivax* cases in 19 Chinese border counties and *P. vivax* prevalence in Myanmar’s five special regions	0.9421 (0.5543–0.9421)	0.8875 (0.3072–0.8875)	<0.01
*Between the number of imported malaria cases in 19 Chinese border counties and parasite prevalence in Myanmar’s five special regions*	*0.9737 (0.7729–0.9737)*	*0.9481 (0.5974–0.9481)*	*<0.001*
Between the number of local *P. falciparum* infections in 19 Chinese border counties and annual *P. falciparum* incidence in Myanmar’s five special regions	0.8098 (−0.0051–0.8098)	0.6558 (0– 0.6558)	>0.05
Between the number of local *P. vivax* infections in 19 Chinese border counties and annual *P. vivax* incidence in Myanmar’s five special regions	0.7632 (−0.1272–0.7632)	0.5824 (0.0162–0.5824)	>0.05
*Between the number of locally infected malaria cases in 19 Chinese border counties and API in Myanmar’s five special regions*	*0.8387 (0.0852–0.8387)*	*0.7035 (0.0073–0.7035)*	*<0.05*
Between the number of local *P. falciparum* infections in 19 Chinese border counties and *P. falciparum* prevalence in Myanmar’s five special regions	0.9404 (0.5442–0.9404)	0.8844 (0.2962–0.8844)	<0.01
Between the number of local *P. vivax* infections in 19 Chinese border counties and *P. vivax* prevalence in Myanmar’s five special regions	0.9535 (0.6273–0.9534)	0.9091 (0.3936–0.9091)	<0.001
*Between the number of locally infected malaria cases in 19 Chinese border counties and parasite prevalence in Myanmar’s five special regions*	*0.9808 (0.8292–0.9808)*	*0.9619 (0.6876–0.9619)*	*<0.001*
Between API in five Chinese counties (YJ, LC, LH, RL and MS) and parasite prevalence in KSR2 of Myanmar	0.9866 (0.8783–0.9866)	0.9734 (0.7714–0.9734)	<0.001
Between API in three Chinese counties (LL, ZK and GM) and parasite prevalence in Kokang of Myanmar	0.9940 (0.9440–0.9940)	0.9881 (0.8912–0.9881)	<0.001
Between API in four Chinese counties (CY, XM, ML and LC) and parasite prevalence in Wa of Myanmar	0.9033 (0.3438–0.9033)	0.8160 (0.1182–0.8160)	<0.01
Between API in three Chinese counties (MH, JH and ML) and parasite prevalence in SR4 of Myanmar	0.8368 (0.0786–0.8368)	0.7002 (0.0062–0.7002)	<0.05

*R* correlation coefficient; *R* ^*2*^ coefficient of determination; *95* *% CI* 95 % confidence interval

## Discussion

The purpose of the paper is to present the relationship of malaria between China’s border side and Myanmar’s border areas. As shown by the results of these linear regressions, API, imported malaria cases and local infections in 19 border counties of China were strongly correlated with parasite prevalence in five special regions of Myanmar, and marginally associated with API of five special regions in Myanmar (Additional file 1; Figs. 2, 3, 4). A sound surveillance system is available for infectious diseases in China, in which malaria cases are reported through CISDCP daily from each public health facility. However, in the five special regions of Myanmar the number of malaria cases is collected and reported only monthly by HPA’s Health Information System, which was established and maintained by the GFATM programmes from 2008 to 2013. The surveillance and reporting system was too weak to collect sufficient information to show the true malaria situation on Myanmar border areas [10]. The weakness included limited capacity of local microscopists, incomplete coverage of surveillance for all communities and the limitation of data reporting and management [6]. In order to overcome the weakness of data collection and reporting system on Myanmar border areas, the sixth round of GFATM programme conducted annual cross-sectional surveys to collect data of parasite prevalence among residents in four of the five special regions of Myanmar, and the tenth round in all five special regions. People with asymptomatic parasites, especially with *P. vivax*, can transmit malaria to others. Investigation of parasite prevalence can detect asymptomatic parasite carriers [13–15]. Based on this evidence, parasite prevalence should show the true malaria situation better than API in Myanmar’s five special regions, to explain the stronger correlations of API on China’s border areas with parasite prevalence on Myanmar’s border.

In 19 Chinese border counties, numbers of both imported and locally infected malaria cases were strongly correlated with parasite prevalence in Myanmar’s five special regions (Additional file 1; Figs. 3, 4). Time from a visit to endemic areas is commonly used to classify cases of imported malaria. Imported cases are defined and classified differently by each country. The WHO recommends administration of a standardized questionnaire to classify the origin of infection in the case of investigation of a malaria case. Based on WHO criterion, travel in an endemic country during the previous 3 years when screening for *P. vivax*, or the previous one year for *P. falciparum*, is defined as an imported malaria case [16]. China uses 1 month from a visit to an endemic country to define and classify imported malaria. If a malaria patient had been in an endemic country more than 1 month previously, the health facility would classify him as a locally infected case in China [5]. This leads to some imported cases being classified into local infections. On the other hand, there are no natural barriers to cross the China–Myanmar border. Some households in a same community belong to China, and others belong to Myanmar, i.e., ‘single village, two countries’. Many of China’s villages and Myanmar communities share the same breeding sites of mosquitoes. *Anopheles* can easily transmit malaria from residents in Myanmar to inhabitants in China. In 2014, Yingjiang County of China reported 17 locally infected malaria cases, 15 (88.2, 95 % CI 63.6–98.5 %) were in villages fewer than 200 m from to Myanmar’s communities. This suggests why the number of local infections on China’s side is so closely correlated with parasite prevalence in Myanmar’s border communities, and indicates the importance of reducing or clearing the parasite reservoir on Myanmar’s side to achieve malaria elimination in the border areas.

From 2008 to 2013, the coefficient of determination (R^2^) between API of 19 Chinese counties and parasite prevalence in five Myanmar’s special regions was 0.9829 (95 % CI 0.8471–0.9829) (Additional file 1; Fig. 2), which means parasite prevalence of five special regions in Myanmar accounted for 98.3 % (95 % CI 84.7–98.3 %) of API in 19 China’s counties, and only 1.7 % (95 % CI 1.7 –15.3 %) of the variation in API of 19 China’s counties was not accounted for by parasite prevalence in Myanmar [7]. When parasite prevalence in KSR2, Kokang, Wa and SR4 of Myanmar was compared to the API in their neighbouring counties of China, the correlation was confirmed (Additional file 1). However, correlation coefficient (R) changed from 0.9940 (95 % CI 0.944–0.994) between three Chinese counties [LL, ZK and GM (Full spelling in Fig. 1)] and Kokang of Myanmar to 0.8368 (95 % CI 0.0786–0.8368) between another three Chinese counties (MH, JH and ML) and SR4 of Myanmar. This shows that other factors, such as climates, landforms and population migration could also influence the malaria relationship between China and Myanmar border areas [17]. One should be careful in interpreting these correlations between API and parasite prevalence in China–Myanmar border areas [7].

The study has four main limitations. First, in total, six cross-sectional surveys were conducted in Myanmar. The sixth round of GFATM programme undertook the first four surveys in four of the five special regions between January and March (dry season, i.e., lower malaria transmission season) in 2008, 2009, 2010 and 2011, respectively. The tenth round of GFATM programme undertook the last two surveys in all the five special regions between September and November (rainy season, i.e., higher transmission season) in 2012 and 2013, respectively. The timing would make a difference in measuring the malaria burden. However, the study was to analyse the relationship between two places, not seasonal difference. The timing difference should not influence the results of the linear regression analysis. Second, the tenth round of China’s GFATM programme operated in KSR1 only in 2012 and 2013, so surveillance data were available for the 2 years only. The relationship between prevalence of KSR1 and its four Chinese neighbouring counties could not be investigated. Third, imported cases are defined and classified differently by country. Malaria patients who visit to endemic countries within 1 month prior to diagnosis were classified as imported cases in China. If a person became infected in China and crossed to Myanmar during the incubation period, was not diagnosed in Myanmar but was sick when he returned to China after a couple of days, the health facility in China would diagnose him as imported malaria. However, classification of imported malaria mainly depends on the difference in malaria endemicity in two countries, not just timing travel. The endemicity of five special regions of Myanmar is much higher than that of 19 counties of China [18]. The timing of the Chinese definition should not influence the results of the study significantly. Fourth, simple linear regressions could not address some potential confounding factors such as socio-economic development, population migration, climate, landforms and deforestation due to unavailability of these data for this study.

## Conclusions

The linear regression analysis documented the strong correlation of malaria between China and Myanmar border areas. This indicates the importance of collaboration on malaria control along the China–Myanmar border for malaria elimination. The stronger correlation of API of China with parasite prevalence than with API in Myanmar’s border areas indicates that parasite prevalence is a better indicator of the true malaria situation in a setting without sound surveillance and reporting system. The strong correlation between locally acquired malaria infection on China’s border and parasite prevalence in Myanmar’s five special regions indicates that the Chinese definition of imported malaria cases should be reconsidered. The definition neglects imported malaria through mosquitoes and asymptomatic parasite carriers on China’s border. The Chinese malaria elimination programme should pay more attention to movement of asymptomatic parasite carriers and imported malaria by mosquitoes. It may be necessary to further strengthen malaria surveillance among border crossers, administrate anti-malarial drugs to people who have been in hyperendemic areas of other countries and plan vector control across the international border [19].

## Additional file

10.1186/s12936-016-1413-4 Malaria along China-Myanmar border.
