# Supplementary material for: The relationship of malaria between Chinese side and Myanmar’s five special regions along China–Myanmar border: a linear regression analysis
Source: Malar J. 2016 Jul 18;15:368. doi: 10.1186/s12936-016-1413-4 (PMC4949750; doi:10.1186/s12936-016-1413-4)
Supplement: Supplementary file 1 — 10.1186/s12936-016-1413-4 Malaria along China-Myanmar border. [file 12936_2016_1413_MOESM1_ESM.doc]

The additional file - data and materials

Table 1 API of China and Myanmar side

|  | 5 special regions(API/10,000) | | | | 19 counties (GS-ML) (API/10,000) | | | |
| --- | --- | --- | --- | --- | --- | --- | --- | --- |
| Year | Person years | Pv | Pf | P spp | Person years | Pv | Pf | P spp |
| 2008 | 195849 | 2765 (141.2, 136.0-146.5) | 5409 (276.2, 269.0-283.5) | 8174 (417.4, 408.6-426.3) | 4603659 | 2235(4.9, 4.7-5.1) | 665(1.4, 1.3-1.6) | 2903(6.3, 6.1-6.5) |
| 2009 | 544876 | 3680 (67.5, 65.4-69.8) | 6738 (123.7, 120.8-126.7) | 10455 (191.9, 188.3-195.6) | 4644719 | 1686(3.6, 3.5-3.8) | 595(1.3, 1.2-1.4) | 2283(4.9, 4.7-5.1) |
| 2010 | 554143 | 2798 (50.5, 48.7-52.4) | 2279 (41.1, 39.5-42.9) | 5084 (91.8, 89.3-94.3) | 4687896 | 1261(2.7, 2.5-2.8) | 570(1.2, 1.1-1.3) | 1833(3.9, 3.7-4.1) |
| 2011 | 187803 | 873 (46.5, 43.5-49.7) | 526 (28.0, 25.7-30.5) | 1413 (75.2, 71.4-79.3) | 4729147 | 732(1.5, 1.4-1.7) | 223(0.5, 0.4-0.5) | 956(2.0, 1.9-2.2) |
| 2012 | 308067 | 1251 (40.6, 38.4-42.9) | 860 (27.9, 26.1-29.8) | 2111 (68.5, 65.6-71.5) | 4770122 | 417(0.9, 0.8-1.0) | 137(0.3, 0.2-0.3) | 556(1.2, 1.1-1.3) |
| 2013 | 626125 | 3181 (50.8, 49.1-52.6) | 1279 (20.4, 19.3-21.6) | 4460 (71.2, 69.2-73.4) | 800944 | 342(0.7, 0.6-0.8) | 81(0.2, 0.1-0.2) | 423(0.9, 0.8-1.0) |

Table 2 Parasitic prevalence on the iMyanmar side

| parasite prevalence(%) of 5 special regions | | | | |
| --- | --- | --- | --- | --- |
| Survey Time | Sample size | Pv | Pf | P spp |
| March, 2008 | 5585 | 460 (8.2, 7.5-9.0) | 299 (5.4, 4.8-6.0) | 761 (13.6, 12.7-14.6) |
| March, 2009 | 3600 | 172 (4.8, 4.1-5.5) | 184 (5.1, 4.4-5.9) | 358 (9.9, 9.0-11.0) |
| March, 2010 | 5090 | 255 (5.0, 4.4-5.6) | 161 (3.2, 2.7-3.7) | 429 (8.4, 7.7-9.2) |
| March, 2011 | 4069 | 69 (1.7, 1.3-2.1) | 44 (1.0, 0.8-1.4) | 113 (2.8, 2.3-3.3) |
| September, 2012 | 4561 | 71 (1.6, 1.2-2.0) | 27 (0.6, 0.4-0.9) | 98 (2.1, 1.7-2.6) |
| November, 2013 | 4517 | 52 (1.2, 0.9-1.5) | 18 (0.4, 0.2-0.6) | 70 (1.5, 1.2-2.0) |

Table 3 The number of local malaria cases and imported malaria cases on China side

| Year | Number and proportion imported | | | Local Number | | |
| --- | --- | --- | --- | --- | --- | --- |
| 2008 | **Vivax (%, 95%CI)** | **Falciparum (%, 95%CI)** | **Total (%, 95%CI)** | **Vivax** | **Falciparum** | **Total** |
| 2009 | 1140(51.0, 48.9-53.1) | 426(64.1, 60.3-67.7) | 1566(53.9, 52.1-55.8) | 1098 | 239 | 1337 |
| 2010 | 1095(65.9, 62.6-67.2) | 429(72.1, 68.2-75.7) | 1524(66.8, 64.8-68.6) | 593 | 166 | 759 |
| 2011 | 881(69.9, 67.2-72.4) | 377(66.1, 62.1-70.0) | 1258(68.6, 66.5-70.8) | 382 | 193 | 575 |
| 2012 | 519(70.9, 67.5-74.2) | 206(92.4, 88.1-95.5) | 734(76.8, 74.0-79.4) | 213 | 17 | 230 |
| 2013 | 283(67.9, 63.1-72.3) | 103(75.2, 67.1-82.2) | 388(69.8, 65.8-73.6) | 135 | 34 | 169 |
| Year | 283(82.7, 78.3-86.6) | 73(90.1, 81.5-95.6) | 356(84.2, 80.3-87.5) | 59 | 8 | 67 |

Table 4 API and parasitic prevalence of KSR2 of Myanmar, and API of 5 counties (YJ, LC, LH, RL, LX) of China,

|  | KSR2 API(API/10,000) | | | | 5 counties (YJ, LC, LH, RL, LX)(API/10,000) | | | | KSR2parasite prevalence(%) | | | | |
| --- | --- | --- | --- | --- | --- | --- | --- | --- | --- | --- | --- | --- | --- |
| Year | Person years | Pv | Pf | P spp | Person years | Pv | Pf | P spp | Survey Time | Sample size | Pv | Pf | P spp |
| 2008 | 58229 | 1889 (324.4, 310.2-339.1) | 3827 (657.2，637.2-677.7) | 5716 (981.6, 957.6-1006.7) | 1167189 | 985(8.4, 6.9-10.3) | 427(3.7, 2.7-5.0) | 1415(12.2, 10.3-14.3) | March, 2008 | 1277 | 89 (7.0, 5.6-8.5) | 129 (10.1，8.5-11.9) | 218 (17.1, 15.0-19.2) |
| 2009 | 59176 | 1178 (199.1, 188.0-210.6) | 2623 (443.3, 426.8-460.2) | 3811 (644.0, 624.4-664.1) | 1177770 | 730(6.2, 4.9-7.8) | 356(3.0, 2.7-3.4) | 1088(9.3, 7.6-11.2) | March, 2009 | 900 | 48 (5.3, 4.0-7.0) | 72 (8.0, 6.3-10.0) | 120 (13.3, 11.2-15.7) |
| 2010 | 60123 | 716 (119.1, 110.6-128.1) | 1223 (203.4, 192.3-215.0) | 1946 (323.7, 309.7-338.1) | 1188416 | 594(5.0, 4.6-5.4) | 396(3.4, 2.4-4.6) | 992(8.3, 6.8-10.2) | March, 2010 | 1110 | 75 (6.8, 5.4-8.4) | 60 (5.4, 4.2-6.9) | 148 (13.3, 11.4-15.5) |
| 2011 | 20362 | 400 (196.4, 177.8-216.5) | 368 (180.7, 162.9-200.2) | 782 (384.1, 358.1-411.4) | 1199019 | 321(2.7, 2.4-3.0) | 141(1.2, 0-2.0) | 462(3.9, 3.5-4.2) | March, 2011 | 1295 | 11 (0.8, 0.4-1.5) | 25 (1.9, 1.3-2.8) | 36 (2.8, 2.0-3.8) |
| 2012 | 31031 | 286 (92.2, 81.8-103.4) | 448 (144.4, 131.4-158.3) | 734 (236.5, 219.9-254.1) | 1209521 | 182(1.5, 1.3-1.7) | 88(0.7, 0.6-0.9) | 270(2.2, 2.0-2.5) | September, 2012 | 1191 | 11 (0.9, 0.5-1.6) | 2 (0.2, 0.02-0.61) | 13 (1.1, 0.6-1.9) |
| 2013 | 63117 | 1339 (212.2, 201.1-223.7) | 435 (68.9, 61.6-75.7) | 1774 (281.1, 268.3-294.3) | 1216870 | 166(1.4, 1.2-1.6) | 50(0.4, 0.3-0.5) | 216(1.8, 1.5-2.0) | November, 2013 | 900 | 10 (1.1, 0.5-2.0) | 2 (0.2, 0.03-0.8) | 12 (1.3, 0.7-2.3) |

Table 5 API and parasitic prevalence of Kongkang of Myanmar, and API of 3 counties (LL, ZK, GM) of China,

|  | KK API(API/10,000) | | | | 3 counties (LL, ZK, GM)(API/10,000) | | | | KK parasite prevalence(%) | | | | |
| --- | --- | --- | --- | --- | --- | --- | --- | --- | --- | --- | --- | --- | --- |
| Year | Person years | Pv | Pf | P spp | Person years | Pv | Pf | P spp | Survey Time | Sample size | Pv | Pf | P spp |
| 2008 | 9872 | 193 (195.5, 169.1-224.8) | 9 (9.1, 4.2-17.3) | 202 (204.6, 177.6-234.5) | 744915 | 283(3.8, 3.3-4.2) | 36(0.5, 0-0.7) | 319(4.3, 3.8-4.8) | March, 2008 | 1400 | 102 (7.3, 6.0-8.8) | 47 (3.4, 2.5-4.4) | 149 (10.6, 9.1-12.4) |
| 2009 | 100323 | 465 (46.4, 42.2-50.7) | 2124 (211.7, 202.9-220.8) | 2589 (258.1, 248.3-268.1) | 753070 | 198(2.6, 2.3-3.0) | 26(0.4, 0-0.5) | 224(3.0, 2.6-3.4) | March, 2009 | 900 | 51 (5.7, 4.2-7.4) | 20 (2.2, 1.4-3.4) | 71 (7.9, 6.2-9.9) |
| 2010 | 101928 | 903 (88.6, 82.9-94.5) | 425 (41.7, 37.8-45.9) | 1328 (130.3, 123.4-137.4) | 761226 | 165(2.2, 1.9-2.5) | 29(0.4, 0-0.6) | 194(2.6, 2.2-2.9) | March, 2010 | 991 | 50 (5.0, 3.8-6.7) | 15 (1.5, 0.8-2.5) | 65 (6.5, 5.1-8.3) |
| 2011 | 34554 | 73 (21.1, 16.6-26.6) | 38 (11.0, 7.8-15.1) | 111 (32.1, 26.4-38.7) | 769390 | 116(1.5, 1.3-1.8) | 11(0.1, 0-0.2) | 127(1.7, 1.4-2.0) | March, 2011 | 963 | 37 (3.8, 2.7-5.3) | 11 (1.1, 0.6-2.0) | 48 (5.0, 3.7-6.6) |
| 2012 | 52712 | 12 (2.3, 1.2-4.0) | 33 (6.3, 4.3-8.9) | 45 (8.5, 6.2-11.4) | 777552 | 56(0.7, 0-0.9) | 6(0.1, 0-0.2) | 62(0.8, 0-1.0) | September, 2012 | 618 | 9 (1.5, 0.7-2.7) | 3 (0.5, 0.1-1.4) | 12 (1.9, 1.0-3.4) |
| 2013 | 107110 | 2 (0.2, 0-0.7) | 5 (0.5, 0-1.1) | 7 (0.7, 0-1.4) | 785714 | 22(0.3, 0-0.4) | 1(0, 0-0.1) | 23(0.3, 0-0.4) | November, 2013 | 900 | 0 (0, 0-0.4) | 0 (0, 0-0.4) | 0 (0, 0-0.4) |

Table 6 API and parasitic prevalence of WA state of Myanmar, and API of 4 counties (CY, XM, ML, LC) of China,

|  | WA API(API/10,000) | | | | 4 counties (CY, XM, ML, LC)(API/10,000) | | | | WA parasite prevalence(%) | | | | |
| --- | --- | --- | --- | --- | --- | --- | --- | --- | --- | --- | --- | --- | --- |
| Year | Person years | Pv | Pf | P spp | Person years | Pv | Pf | P spp | Survey Time | Sample size | Pv | Pf | P spp |
| 2008 | 27880 | 210 (75.3, 65.5-86.2) | 135 (48.4, 40.6-57.3) | 345 (123.7, 111.1-137.4) | 868251 | 206(2.4, 2.1-2.7) | 44(0.5, 0-0.7) | 250(2.9, 2.5-2.3) | March, 2008 | 1535 | 175 (11.4, 9.9-13.1) | 84 (5.5, 4.4-6.7) | 261 (17.0, 15.2-19.0) |
| 2009 | 283329 | 1670 (58.9, 56.2-61.8) | 1583 (55.9, 53.2-58.7) | 3280 (115.8, 111.9-119.8) | 878588 | 109(1.2, 1.0-1.5) | 27(0.3, 0-0.4) | 136(1.6, 1.3-1.8) | March, 2009 | 900 | 39 (4.3, 3.1-5.9) | 39 (4.3, 3.1-5.9) | 80 (8.9, 7.1-10.9) |
| 2010 | 288146 | 938 (32.6, 30.5-34.7) | 470 (16.3, 14.9-17.9) | 1408 (48.9, 46.4-51.5) | 890092 | 56(0.6, 0-0.8) | 9(0.1, 0-0.2) | 65(0.7, 0-0.9) | March, 2010 | 1697 | 60 (3.5, 2.7-4.5) | 80 (4.7, 3.8-5.8) | 140 (8.2, 7.0-9.6) |
| 2011 | 97585 | 333 (34.1, 30.6-38.0) | 110 (11.3, 9.3-13.6) | 443 (45.4, 41.3-49.8) | 900529 | 52(0.6, 0-0.8) | 12(0.1, 0-0.2) | 64(0.7, 0-0.9) | March, 2011 | 900 | 8 (0.9, 0.4-1.7) | 7 (0.8, 0.3-1.6) | 15(1.7, 0.9-2.7) |
| 2012 | 148867 | 226 (15.2, 13.3-17.3) | 36 (2.4, 1.7-3.4) | 262 (17.6, 15.5-19.9) | 910966 | 27(0.3, 0-0.6) | 3 (0, 0-0.1) | 30(0.3, 0-0.5) | September, 2012 | 949 | 14 (1.5, 0.8-2.5) | 1 (0.1, 0-0.6) | 15 (1.6, 0.9-2.6) |
| 2013 | 302496 | 211 (7.0, 6.1-8.0) | 49 (1.6, 1.2-2.2) | 260 (8.6, 7.6-9.7) | 914466 | 15(0.2, 0-0.5) | 7(0.1, 0-0.2) | 22(0.2, 0-0.4) | November, 2013 | 900 | 0 (0, 0-0.4) | 0 (0, 0-0.4) | 0 (0, 0-0.4) |

Table 7 API and parasitic prevalence of SR4 of Myanmar, and API of 3 counties (MH, JH, MLa)of China.

|  | SR4 API(API/10,000) | | | | 3 counties (MH, JH, MLa)(API/10,000) | | | | SR4 parasite prevalence(%) | | | | |
| --- | --- | --- | --- | --- | --- | --- | --- | --- | --- | --- | --- | --- | --- |
| Year | Person years | Pv | Pf | P spp | Person years | Pv | Pf | P spp | Survey Time | Sample size | Pv | Pf | P spp |
| 2008 | 99868 | 473 (47.4, 43.2-51.8) | 1438 (144.0, 136.7-151.6) | 1911 (191.4, 183.0-200.0) | 1027688 | 111(1.1, 0.9-1.3) | 20(0.2, 0.1-0.3) | 131(1.3, 1.1-1.5) | March, 2008 | 1373 | 94 (6.8, 5.6-8.3) | 39 (2.8, 2.0-3.9) | 133 (9.7, 8.2-11.4) |
| 2009 | 1E+05 | 367 (36.0, 32.4-39.8) | 408 (40.0, 36.2-44.1) | 775 (75.9, 70.7-81.5) | 1034920 | 43(0.4, 0.3-0.6) | 6(0.1, 0.0-0.1) | 49(0.5, 0.4-0.6) | March, 2009 | 900 | 34 (3.8, 2.6-5.2) | 53 (5.9, 4.4-7.6) | 87 (9.7, 7.8-11.8) |
| 2010 | 1E+05 | 241 (23.2, 20.4-26.3) | 161 (15.5, 13.2-18.1) | 402 (38.7, 35.0-42.6) | 1042130 | 25(0.2, 0.2-0.4) | 9(0.1, 0-0.2) | 34(0.3, 0.2-0.5) | March, 2010 | 1292 | 70 (5.4, 4.2-6.8) | 6 (0.5, 0.2-1.0) | 76 (5.9, 4.7-7.3) |
| 2011 | 35303 | 67 (19.0, 14.7-21.1) | 10 (2.8, 1.4-5.2) | 77 (21.8, 17.2-27.3) | 1049379 | 14(0.1, 0-0.2) | 3(0.1, 0-0.1) | 17(0.2, 0-0.3) | March, 2011 | 910 | 13 (1.4, 0.8-2.4) | 1 (0.1, 0-0.6) | 14 (1.5, 0.8-2.6) |
| 2012 | 53730 | 88 (16.4, 13.1-20.2) | 11(2.1, 1.0-3.7) | 99 (18.4, 15.0-22.4) | 1056628 | 14(0.1, 0-0.2) | 3(0.1, 0-0.1) | 17(0.2, 0-0.3) | September, 2012 | 902 | 8 (0.9, 0.4-1.7) | 4 (0.4, 0.1-1.1) | 12 (1.3, 0.7-2.3) |
| 2013 | 1E+05 | 293 (26.8, 23.9-30.1) | 55 (5.0, 3.8-6.6) | 348 (31.9, 28.6-35.4) | 1063877 | 13(0.1, 0-0.2) | 0(0, 0-0.03) | 13(0.1, 0-0.2) | November, 2013 | 900 | 12 (1.3, 0.7-2.3) | 3 (0.3, 0.1-1.0) | 15 (1.7, 0.9-2.7) |
